# Critical care delivery models in emergency departments: a systematic review of the literature and meta-analysis of related outcome effects

**DOI:** 10.1186/s13049-026-01647-9

**Published:** 2026-06-14

**Authors:** Matthias Noitz, Steven A. Ilko, Martin W. Dünser, Wilhelm Behringer, Brigitte Wildner, Harald Herkner

**Affiliations:** 1 Department of Anesthesiology and Critical Care Medicine, Kepler University Hospital GmbH, Johannes Kepler University Linz, Krankenhausstraße 9, 4020 Linz and Altenberger Strasse 69, Linz, 4040 Austria; 2 Medical Faculty, Johannes Kepler University, Linz, Austria; 3 Department of Emergency Medicine Center for Adult Critical Care, University of New Mexico, Albuquerque, NM USA; 4 Department of Anesthesia, University of Iowa Healthcare, Iowa City, IA USA; 5 Department of Emergency Medicine, Medical University of Vienna, Vienna, Austria; 6 University Library, Medical University of Vienna, Vienna, Austria

**Keywords:** Critical care delivery models, Emergency department, Intensive care unit, Emergency critical care, Intensive care unit admission, Mortality

## Abstract

**Background:**

Critical care delivery in the emergency department (ED) may improve selected patient- and system-relevant outcomes. However, it remains unclear which organisational models to deliver critical care in the ED (CC-ED) have been implemented and what their outcome effects are.

**Methods:**

We conducted a systematic review and meta-analysis including studies describing organisational approaches to deliver CC-ED. Delivery models were categorised and described qualitatively. For studies reporting comparable outcomes, random-effects meta-analysis was performed to estimate pooled effects and 95% confidence intervals. Outcomes included mortality, intensive care unit (ICU) admission rates, ICU length of stay (LOS), hospital LOS, and cost of care.

**Results:**

Of 4,967 records, 67 were included into the qualitative assessment and 11 studies into the quantitative analysis, respectively. We identified five models to deliver CC-ED. These consisted of dedicated critical care areas in the ED (*n* = 49/67, 73.1%), placement of critical care staff in the ED (*n* = 5/67, 7.5%), deployment of critical care teams to the ED (*n* = 5/67, 7.5%), telemedical support of ED staff by critical care teams (*n* = 5/67, 7.5%), and implementation of protocols to accelerate ICU admission (*n* = 3/67, 4.5%). Mortality was not different versus usual care in any of the CC-ED models. CC-ED models had variable effects on the ICU admission rate, hospital and ICU LOS. Dedicated critical care areas in the ED were associated with lower ICU admission rates [OR 0.83 (0.76–0.91), *p* < 0.001], and shorter hospital LOS [mean difference − 0.31 (-0.59 to -0.03) days, *p* = 0.03], but longer ICU LOS [mean difference 0.45 (0.31–0.6) days, *p* < 0.001]. Reported cost of care did not differ between CC-ED and usual care.

**Conclusions:**

We identified five different organisational model categories to deliver CC-ED. Dedicated critical care areas such as ED-ICUs was the model category most frequently published. Our quantitative meta-analysis suggests that compared with other CC-ED delivery models, dedicated critical care areas in the ED may reduce both ICU admission rates and hospital LOS of critically ill ED patients.

**Supplementary Information:**

The online version contains supplementary material available at 10.1186/s13049-026-01647-9.

## Background

Critical illness is a time sensitive continuum of diseases requiring rapid medical interventions to stabilise vital organ functions and reverse the underlying condition [1]. Critically ill patients make up an important group of patients presenting to the emergency department (ED) [2]. Large studies demonstrated that prolonged time spent in the ED prior to intensive care unit (ICU) admission was associated with higher mortality and longer ICU as well as hospital length of stays (LOS) in critically ill patients [3–7]. However, this association between ED LOS and mortality disappeared, when early and appropriate critical care was performed in the ED rather than being delayed until ICU admission [8, 9]. A recent meta-analysis of predominantly urban academic centres with established critical care delivery models in the ED, found no significant association between ED boarding time and mortality or hospital LOS [10]. Critical care interventions can rapidly stabilise organ functions, halt or reverse organ dysfunction, and in some cases even avoid the need for ICU admission [2, 11]. The most important benefits of providing timely critical care in the ED are a reduced rate of ICU admissions among critically ill ED patients, shorter ICU and hospital lengths of stay, increased ICU capacities for non-ED patients, as well as improved short- and long-term survival [2, 11]. A European group of emergency and critical care physicians has recently emphasized the importance and need for timely critical care delivery in the ED, highlighting that the ED treatment phase was an integral part in the continuum of care for critically ill patients [11]. Different concepts, models and solutions on how to provide critical care in the ED and improve the management of critically ill ED patients have been published in the past [12–14]. It remains unknown, which organisational models to deliver critical care in the ED have so far been implemented and what their effects on patient-relevant outcomes are.

This systematic review aimed to identify and describe all published organisational models for critical care delivery in the ED. In addition, we performed a meta-analysis to quantify the effects of these ED-based critical care delivery models on the ICU admission rate, ICU and hospital LOS, as well as mortality.

## Methods

This evidence synthesis was conducted according to a pre-published protocol, which had been registered at an international systematic review registry (PROSPERO; https://www.crd.york.ac.uk/PROSPERO/view/CRD42024517246; Date of registration: March 16th, 2024). The 2020 Preferred Reporting Items for Systematic Reviews and Meta-analyses (PRISMA) statement guided the drafting of this manuscript [15].

### Study objectives

This evidence synthesis had two study objectives. The first objective was to identify and qualitatively describe all organisational models to deliver critical care in the ED, which have so far been published in the literature. The second objective was to quantify the effects of these models on the ICU admission rate, ICU and hospital LOS, as well as mortality using a quantitative meta-analysis. It was not an objective of this evidence synthesis to assess the effects of critical care delivery outside of the ED (e.g., critical care delivery in the pre-hospital setting, critical care delivery on hospital wards).

### Criteria for considering studies for this review

#### Type of studies

Original, peer-reviewed research articles describing emergency department-based critical care delivery models were eligible for inclusion. These predominantly comprised prospective or retrospective cohort studies, prospective or retrospective before-and-after studies, stepped-wedge studies, and cluster randomized trials. We excluded case reports and case series enrolling < 10 patients, study protocols, narrative reviews, articles published only as abstracts or poster presentations, and publications in non-peer-reviewed journals.

#### Type of participants

Only studies involving patients treated in the ED were eligible for inclusion. All age groups were considered. In view of different global organisational structures, EDs were broadly defined as the receiving areas of hospitals, where unscheduled patients or those brought in by emergency medical services because of an acute disease are initially cared for.

#### Type of interventions

We defined organisational models for delivering critical care in the ED as modifications or adoptions of ED organisation, specifically aiming to improve the care of critically ill patients. Medical protocols to guide the therapy of critically ill patients or specific critical illnesses (e.g., sepsis) in the ED were not regarded as organisational models. Models of critical care delivery outside the ED (e.g., prehospital settings or inpatient wards) were not included. For the quantitative meta-analysis, usual ED care, as defined by the study authors, served as the comparator.

#### Types of outcomes

Primary outcomes of the quantitative meta-analysis included the reported, unadjusted all-cause mortality closest to day 28 after ED admission and health-related quality of life of ED patients. Secondary outcomes of the quantitative meta-analysis included the ICU admission rate, ICU LOS, hospital LOS, and cost of care.

### Search methods for identification of studies

The following electronic databases were used as information sources: CENTRAL Cochrane Central Register of Controlled Trials (Ovid), MEDLINE (Ovid), and EMBASE (https://www.embase.com/). Both defined vocabulary (e.g., medical subject headings) and free-text terms were used as search terms (Electronic Supplementary Material Table E1). Additionally, we searched for relevant systematic reviews on the topic in Epistemonikos (https://www.epistemonikos.org/). Bibliographic references and citations of both included studies and identified reviews were screened to identify other potentially eligible studies. The literature search was conducted in each electronic database from inception until March 5, 2024. The final search update was performed on March 5, 2024.

### Study selection and data extraction

#### Selection of studies

After completion of the literature search, all identified references were uploaded into a bibliographic software (Covidence, Melbourne, Australia; https://www.covidence.org/). Duplicates were automatically removed. Two authors (MN, SAI) independently screened all publications. In a first step, publications were screened by title and abstract for relevance. Subsequently, full texts as identified by the title and abstract search were reviewed by the same pair of authors. If the full-texts could not be retrieved by efforts including contacting the corresponding author, the study was marked as ‘unable to retrieve full-text’. Following an in-depth analysis of full-texts and application of eligibility criteria, the agreement of both authors was necessary to include the publication into both the qualitative and quantitative analysis. In case of disagreement, consensus was achieved by discussion or decision making by a third author (HH or MWD). Reasons for full-text exclusion were documented.

#### Data extraction

Data from selected studies were extracted using an electronic data extraction form. The development of this spreadsheet was informed by pilot testing and discussion among the authors. Extracted data included study and participant characteristics, country of origin, hospital level, information about the intervention (organisational model to deliver critical care in the ED) and control group (usual care), primary and secondary outcome measures, items on risk of bias, and (whenever available) subgroup identifiers. Two review authors (MN, SAI) independently extracted all data, entered these into the data extraction form and subsequently compared their entries. One review author (MN) then transferred the data into the final datasheet for analysis, with the other review author (SAI) double-checking the process. In case of missing data, we attempted to obtain the information directly from the study authors.

#### Risk of bias assessment

The methodological quality and risk of bias of individual studies included into the quantitative meta-analysis were evaluated using the Joanna Briggs Institute tools [16]. The risk of bias assessment was performed independently by two authors (MN, SAI). Disagreement was solved by discussion or involvement of a third author (HH or MWD). Risk of bias items for each study were entered into the electronic spreadsheet, as outlined above.

### Data synthesis and meta-analysis

All extracted data were first displayed in tabular format. A qualitative synthesis of extracted data was prepared to report the different organisational model categories to deliver critical care in the ED. For the quantitative synthesis all studies were systematically assessed for both clinical and methodological homogeneity by content and methodological expertise (HH, WB, MWD). We had decided to synthesise before-and-after studies, studies applying a stepped-wedge design, and cluster randomized trials, but refrained from synthesising results of concurrent cohort studies where an uncontrolled selection process led to the choice between care models, because these study designs did not allow to analyse comparable patient groups. In case multiple publications reported data from the same underlying patient population, we carefully assessed overlap in study periods, populations, outcomes, and intervention definitions. To avoid unit-of-analysis errors, publications were not pooled across reports unless complete comparability could be ensured. Where overlap or relevant clinical or methodological differences were identified, the most informative non-overlapping publication was selected for quantitative synthesis, while remaining reports were analysed separately or retained for qualitative synthesis. Only in the absence of relevant clinical or methodological heterogeneity we decided to pool outcome effects. Assuming some underlying heterogeneity throughout we decided to use random effects models as the default. We calculated point estimates of odds ratios or mean differences with 95% confidence intervals (95% CI). For cost outcomes, random-effects meta-analysis using restricted maximum likelihood estimation with Hartung–Knapp adjustment was performed. No imputation methods were used to compensate for missing data. Statistical heterogeneity was quantified using the *I²*-statistics. Whenever a sufficient number of studies could be identified, sensitivity analyses according to risk of bias items were conducted. As post-hoc sensitivity analyses we used adjusted effect estimates from studies where these were available, instead of using the unadjusted estimates. Finally, the Grading of Recommendations Assessment, Development and Education (GRADE) methodology was used to determine the overall quality of evidence supporting the results of the quantitative meta-analysis.

## Results

**Fig. 1:**
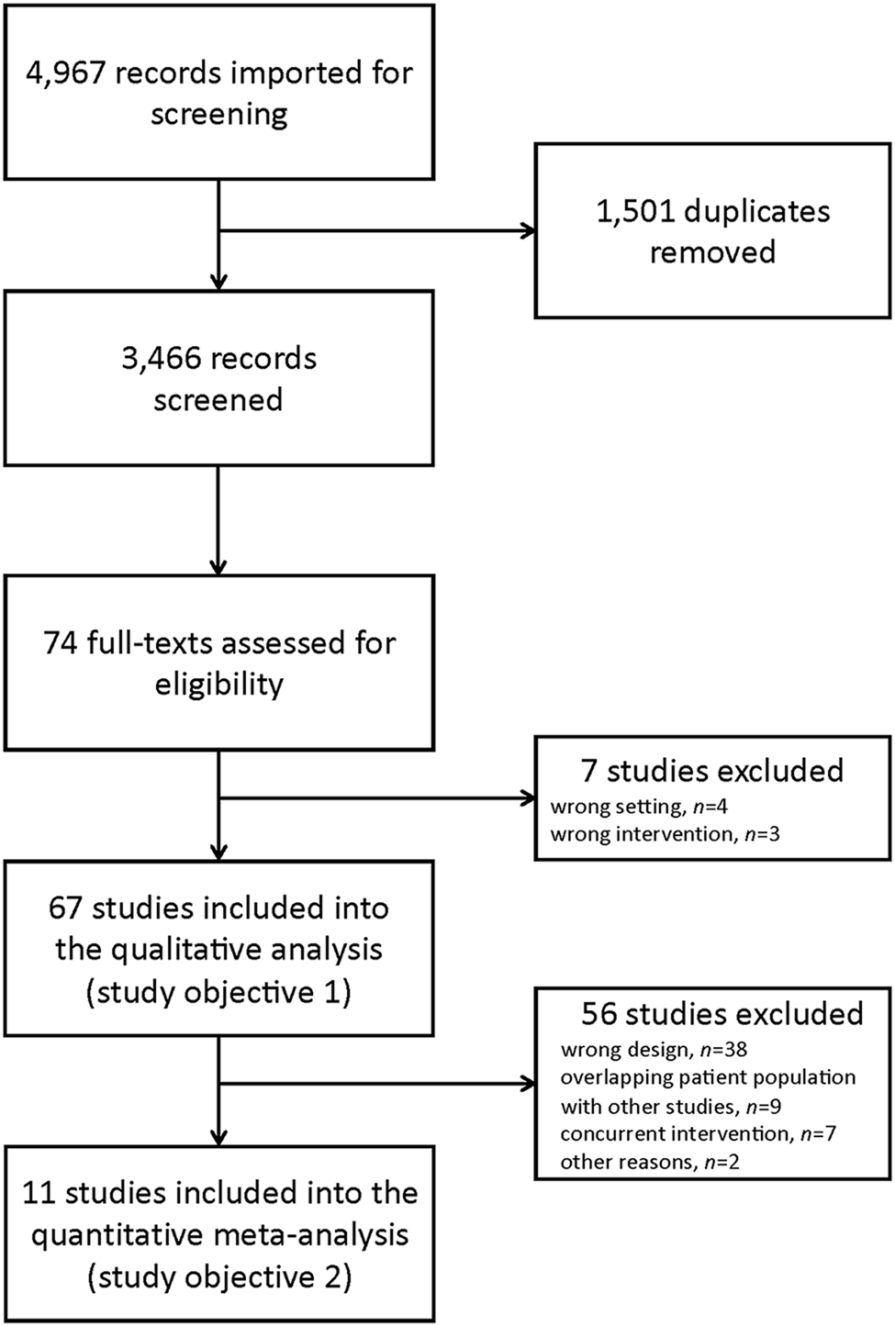
Study flow diagram

Out of 4,967 records identified through the database search, we included 67 studies into the qualitative analysis and 11 studies into the quantitative analysis, respectively (Fig. 1).

### Qualitative analysis of organisational models to deliver critical care in the ED

**Fig. 2:**
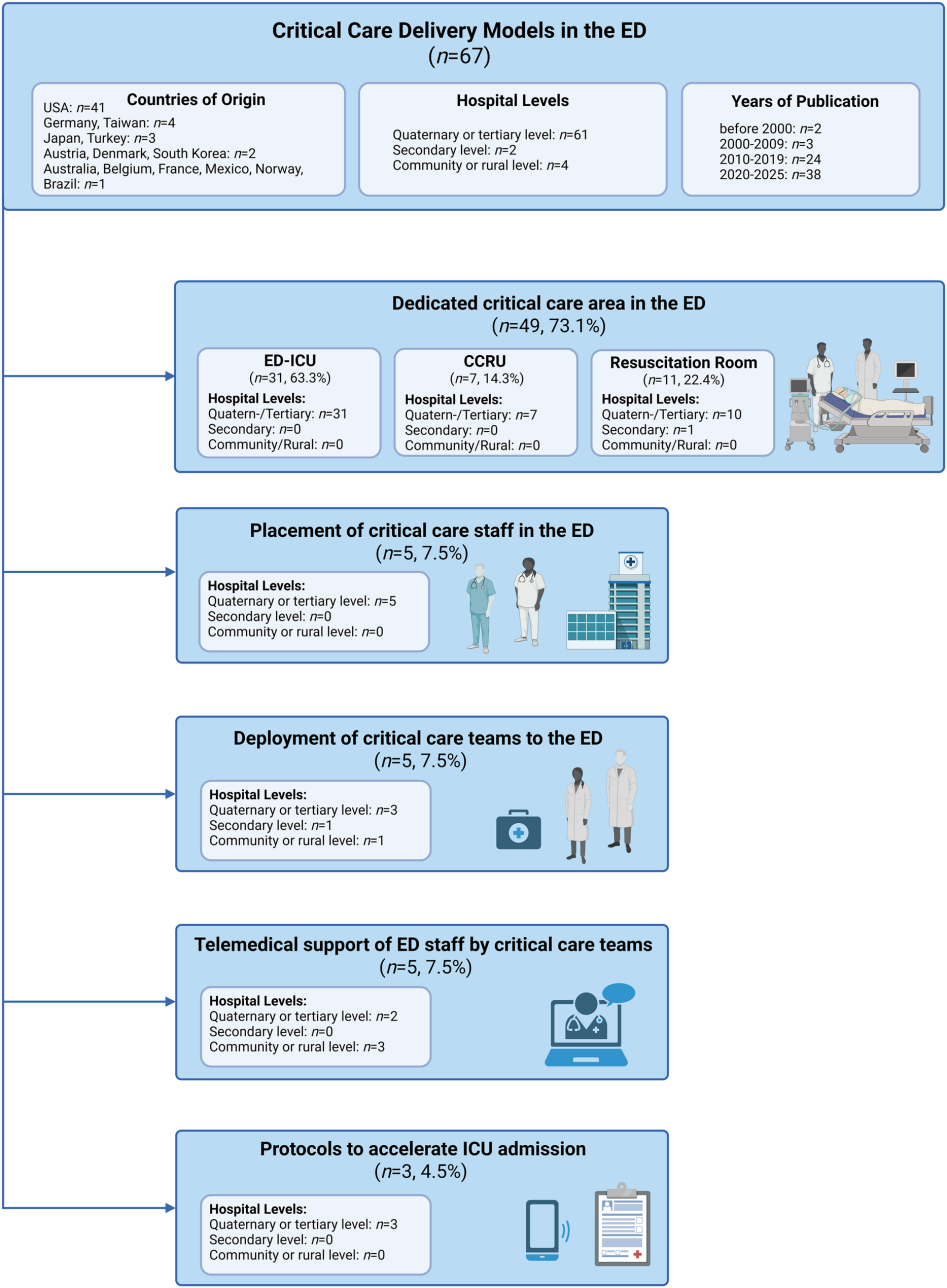
Overview of the five published organisational model categories to deliver critical care in the emergency department as identified by the systematic review of the literature. CCRU, critical care resuscitation unit; ED, emergency department; ED-ICU, intensive care unit located in the emergency department; ICU, intensive care unit

Of the 67 included studies, 36 (53.7%) were retrospective cohort studies, 20 (29.9%) were before–after studies, 4 (6.0%) were prospective cohort studies, 3 (4.5%) were cross-sectional/descriptive studies, and 4 (6.0%) were classified as other observational or descriptive study designs. Collectively, these studies represented 39 unique units or locations. Five different organisational model categories to deliver critical care in the ED were reported in the 67 studies and included in the qualitative analysis (Fig. 2; Table 1). Forty-one studies (61.2%) originated from the United States, twelve (17.9%) from Asia, eleven (16.4%) from Europe, and one (1.5%) each from Australia, Central America, and South America. Nine centres published their models two or more times (Ann Arbor/USA, *n* = 14; Baltimore/USA, *n* = 7; Philadelphia/USA,* n *= 3; Shimane/Japan, *n* = 3; Dallas/USA, *n* = 2; Leipzig/Germany, *n* = 2; Stanford/USA, *n* = 2; Taipei/Taiwan, *n* = 2; Vienna/Austria, *n* = 2). A dedicated critical care area in the ED was the most frequently published model to deliver critical care in the ED (*n* = 49/67, 73.1%) [17–64]. This critical care delivery model category included ICUs located in the ED (ED-ICUs) (*n* = 31) [8–31, 34–39, 42–44, 48, 49, 51–53, 57–64], critical care resuscitation units (CCRUs) (*n* = 7) [21, 22, 28, 32, 40, 41, 50], and resuscitation rooms (*n* = 11) [19, 20, 24, 27, 33, 45–47, 54–56]. Further model categories to deliver critical care in the ED comprised placement of critical care staff in the ED (*n* = 5/67, 7.5%) [65–69], deployment of critical care teams to the ED in case a critically ill patient was identified (*n* = 5/67, 7.5%) [70–74], telemedical support of ED staff by critical care teams (*n* = 5/67, 7.5%) [75–79], and implementation of protocols to accelerate ICU admission (*n* = 3/67, 4.5%) [80–82]. Sixty-three studies (94%) reported organisational models to deliver critical care in adult EDs, while four studies (6%) specifically described organisational models to deliver critical care in paediatric EDs. When classified by unique units or locations rather than publications, dedicated critical care areas in the ED remained the most common model (19 units), followed by deployment of critical care teams (5 units), placement of critical care staff in the ED (4 units), telemedical support models (3 units), and protocols to accelerate ICU admission (3 units). Among the 19 unique dedicated critical care areas identified, unit size was reported for 16 (84.2%) units and ranged from 1 to 17 beds/treatment spaces [median number of beds 8, IQR (6–13)]. Nine units served a broad critically ill ED population, whereas eight were restricted to selected patient populations. Eligibility criteria for CC-ED treatment were unclear or not reported for two units.

Critical care competencies of physician staffing were specified in 65 (97%) studies. Fifty-three of them (81.5%) reported that physicians were trained in critical care, while physicians were dually trained in emergency and critical care medicine in 31 records (47.7%). Thirty-seven (75.5%) and 28 (57.1%) of the 49 studies describing dedicated critical care areas in the ED reported involvement of critical care-trained physicians and physicians dually trained in emergency and critical care medicine, respectively. Overall, 52 of 67 publications (77.6%) from 24 individual study sites reported information on nurse staffing, either directly or via complementary reports from the same site. Of these, 37 (71.2%) studies from 13 centres indicated that nurses were trained in critical care. Forty-three (64.2%) publications described organisational models that were operational on a 24-hours/7-days basis, while ten (14.9%) operated only during selected time periods. Operational hours were not specified in fourteen (20.9%) reports.

### Quantitative synthesis of the effects of organisational model categories to deliver critical care in the ED

**Fig. 3:**
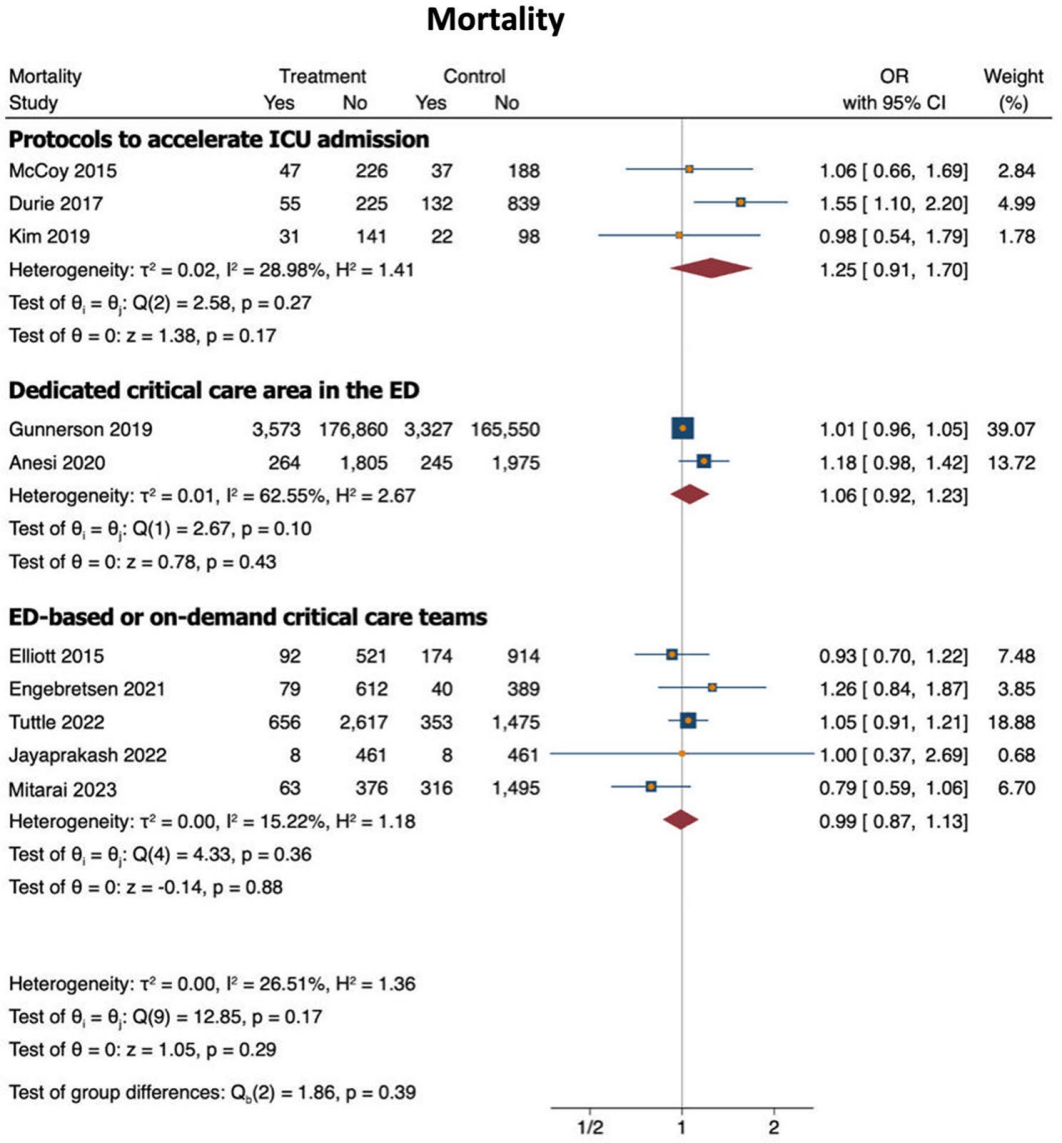
Pooled effects of different organisational model categories to deliver critical care in the emergency department on mortality of critically ill emergency department patients. ED, emergency department; ED-ICU, intensive care unit located in the emergency department; ICU, intensive care unit

Eleven studies reporting different organisational model categories to deliver critical care in the ED [dedicated critical care area in the ED [23, 34, 42], *n* = 3; ED-based or on-demand critical care teams, *n* = 5 [65, 67, 68, 71, 72]; protocols to accelerate ICU admission, *n* = 3 [80–82]] were selected for the quantitative meta-analysis (Fig. 1). All analyses included were retrospective cohort studies mostly applying before-and-after comparisons. Specific details of study interventions are outlined in Table 1. Usual care in control groups represented patient care before organisational models to deliver critical care in the ED had been implemented or during periods when these organisational models were unavailable. The risk of bias was rated as low in four [23, 42, 65, 72] and high in seven [34, 67, 68, 71, 80–82] studies. Detailed risk-of-bias assessments for studies included in the quantitative synthesis are provided in Electronic Supplementary Figure E1. All-cause mortality was reported in ten studies. In the quantitative analysis, no significant difference in unadjusted mortality was found across any of the organisational models investigated compared with usual care (Fig. 3). Health-related quality of life was prespecified in the published study protocol as a primary outcome. However, indicators of health-related quality of life were not reported in any of the studies included and therefore not analysed.

**Table 1:** Detailed description of identified organisational models to deliver critical care in the emergency department

First Author, Year (Ref)	City, Country	Hospital Level	Model Type	Detailed Description	Staffing	Operational Hours
**Dedicated critical care area in the ED**						
Ayvali, 2023 [17]	Ankara, Turkey	Tertiary	ED-ICU	Emergency critical care unit (ECCU) with 8 beds in the ED	Emergency medicine specialist and assistants	not given
Doan, 2023 [18]	Ann Arbor, USA	Quaternary	ED-ICU	see Gunnerson, 2019 [42]		
Dziegielew-ski, 2023 [19]	Duesseldorf, Germany	Tertiary	Resuscitation Room	Four specially equipped resuscitation rooms dedicated to the care of critically ill non-trauma and trauma patients in the ED.	2 nurses, 1 resident and 1 attending physician with emergency and critical care competencies, other specialists consulted as required	24-hours/7-days
Matsumoto, 2023 [20]	Shimane, Japan	Tertiary	Hybrid Emergency Room	see Watanabe, 2018 [47]		
Tran, 2023 [21]	Baltimore, USA	Quaternary	Critical Care Resuscitation Unit (CCRU)	see Scalea, 2016 [50]		
Tran, 2023 [22]	Baltimore, USA	Quaternary	Critical Care Resuscitation Unit (CCRU)	see Scalea, 2016 [50]		
Bassin, 2022 [23]	Ann Arbor, USA	Quaternary	ED-ICU	see Gunnerson, 2019 [42]		
Grahl, 2022 [24]	Leipzig, Germany	Tertiary	Resuscitation room	Resuscitation room in the ED	2 nurses, 1 resident and 1 attending physician with emergency and critical care competencies	24-hours/7-days
Haas, 2022 [25]	Ann Arbor, USA	Quaternary	ED-ICU	see Gunnerson, 2019 [42]		
Harvey, 2022 [26]	Ann Arbor, USA	Quaternary	ED-ICU	see Gunnerson, 2019 [42]		
Kreß, 2022 [27]	Mönchengladbach, Germany	Tertiary	Resuscitation room	3 resuscitation rooms in the ED. All resuscitation rooms have standard equipment according to the airway, breathing, circulation, disability and environment scheme, video laryngoscopy, point of care blood gas analysis, mobile sonography units and mechanical chest compression devices.	Basic team: 1 ED nurse, 1–2 ED physicians Expanded team: 1 ED resident, 1 ED consultant, 2 ED nurses Cardiac arrest receiving team: 2 ED residents, 1 ED consultant, 2 ED nurses, cardiology consultant and orthopaedic surgery resident/consultant on demand	24-hours/7-days
Miller, 2022 [28]	Baltimore, USA	Quaternary	Critical Care Resuscitation Unit (CCRU)	see Scalea, 2016 [50]		
Puls, 2022 (8)	Ann Arbor, USA	Quaternary	ED-ICU	see Gunnerson, 2019 [42]		
Du, 2021 [29]	Ann Arbor, USA	Quaternary	ED-ICU	see Gunnerson, 2019 [42]		
Guven, 2021 [30]	Istanbul, Turkey	Tertiary	ED-ICU	ED-ICU is an isolated separate unit located near the emergency service main area.	Primarily managed by ED physicians. ED physicians working in ED-ICU have attended critical care courses and have critical care certificates. Doctors working in the ED-ICU are only responsible for this area in ED-ICU shifts.	not given
Haas, 2021 [31]	Ann Arbor, USA	Quaternary	ED-ICU	see Gunnerson, 2019 [42]		
Tiffany, 2021 [32]	Baltimore, USA	Quaternary	Critical Care Resuscitation Unit (CCRU)	see Scalea, 2016 [50]		
Watanabe, 2021 [33]	Shimane, Japan	Tertiary	Hybrid Emergency Room	see Watanabe, 2018 [47]		
Anesi, 2020 [34]	Philadelphia, USA	Quaternary	ED-ICU (ResCCU)	Five-bed multidisciplinary ICU embedded in the ED. All ED patients were initially managed by ED physicians. All critically ill non-trauma patients requiring > 1 h of critical care were rapidly transferred to the ED-ICU (even if an inpatient ICU bed was available)	ED physicians, emergency medicine-trained intensivists, ED-/ICU-trained nurses, respiratory therapists, clinical pharmacists	Monday 7.00 am to Saturday 7.00 am
Haas, 2020 [35]	Ann Arbor, USA	Quaternary	ED-ICU	see Gunnerson, 2019 [42]		
Haas, 2020 [36]	Ann Arbor, USA	Quaternary	ED-ICU	see Gunnerson, 2019 [42]		
Jeong, 2020 [37]	Seoul, South Korea	Tertiary	ED-ICU	ED-ICU received only ED patients. ED-ICU physician notified as soon as critically ill ED patient was identified. ED-ICU physician provided initial resuscitation and admitted patients to ED-ICU or other ICUs (in case of no available bed or specialty care required)	ED physicians certified as intensivists (2 years of critical care fellowship), nurses (nurse: patient ratio, 1:2)	24-hours/7-days
Joseph, 2020 [38]	Ann Arbor, USA	Quaternary	ED-ICU	see Gunnerson, 2019 [42]		
Mudan, 2020 [39]	Philadelphia, USA	Quaternary	ED-ICU (ResCCU)	Six-bed ED-based intensive care unit able to deliver ICU level care beyond the initial resuscitation period within the ED.	Critical care-trained ED physicians. Specialized nurses with a maximum nursing ratio of 2 patients to 1 nurse.	24-hours/ Monday to Friday
Tran, 2020 [40]	Baltimore, USA	Quaternary	Critical Care Resuscitation Unit (CCRU)	see Scalea, 2016 [50]		
Tran, 2020 [41]	Baltimore, USA	Quaternary	Critical Care Resuscitation Unit (CCRU)	see Scalea, 2016 [50]		
Gunnerson, 2019 [42]	Ann Arbor, USA	Quaternary	ED-ICU	7,800 square foot unit (emergency critical care centre) consisting of 5 resuscitation or trauma bays and 9 ICU rooms adjacent to the main ED. Only critically ill ED patients (no specific criteria for definition) were admitted to the ICU rooms from resuscitation bays or ED treatment rooms (even if an inpatient ICU bed was available).	ED physicians with or without critical care training, residents, physician assistants, ED nurses with critical care experience (nurse: patient ratio, 1:2), respiratory therapists, pharmacists	24-hours/7-days
Wang, 2019 [43]	Taipei, Taiwan	Tertiary	ED-ICU	ED-ICU capable of providing same level of care as the medical and/or surgical ICU including advanced haemodynamic monitoring, vasopressor therapy, ventilator support, continuous renal replacement therapy, extracorporeal membrane oxygenation support, and post-cardiac arrest care.	Physicians dually trained in emergency and critical care medicine.	not given
Zhou, 2019 [44]	Philadelphia, USA	Tertiary	ED-ICU (ResCCU)	5- to 6-bed ED-based ICU	Critical care trained emergency physicians, nurses in a 1:1–2 nurse: patient ratio	Monday to Friday
Bernhard, 2018 [45]	Leipzig, Germany	Tertiary	Resuscitation Room	ED resuscitation room for the management of critically ill non-trauma patients	Team of two nurses, one resident and one senior physician with emergency and critical care competencies	24-hours/7-days
Chavez, 2018 [46]	Memorial, USA	Tertiary	Medical Resuscitation Bay	Resuscitation bay in a paediatric ED	Paediatric emergency-trained physicians, with additional support provided by paediatricians	24-hours/7-days
Watanabe, 2018 [47]	Shimane, Japan	Tertiary	Hybrid Emergency Room	Large resuscitation room in the ED, which is equipped with critical care equipment, a computed tomography and angiography system able to provide full resuscitation, radiographic imaging, endovascular treatment and emergency surgery including damage control surgery without the need to transfer the patient.	Interdisciplinary staffing including trauma surgeons, a trauma nurse, a radiologist, an interventionist, and emergency physicians.	24-hours/7-days
Fu, 2017 [48]	Taipei, Taiwan	Tertiary	ED-ICU	ICU containing 13 beds and located in the ED. The ED-ICU receives critically ill ED patients who cannot be admitted to a specialized ICU immediately after initial ED resuscitation and stabilisation. It operates in a semi-open model that both ED physicians and cardiologists take care of all patients with cardiovascular emergencies.	ED physicians board-certified in critical care, cardiologists, emergency physicians and residents, nurses (nurse: patient ratio, 1:2)	24-hours/7-days
Puy, 2017 [49]	Amiens, France	Tertiary	ED-ICU	ICU with 6 rooms located in the ED. The unit was created to manage general critical care patients and stroke patients admitted through the ED.	Emergency physicians, nurses, nursing assistants with experience of life-threatening emergencies and training in stroke management	24-hours/7-days
Scalea, 2016 [50]	Baltimore, USA	Quaternary	Critical Care Resuscitation Unit (CCRU)	CCRU with 6 ICU beds to facilitate immediate ICU access and accommodate interhospital transfers of patients with time-sensitive surgical critical illness from external EDs or hospitals. CCRU length of stay goal was < 6 h.	Attending intensivist. 3 to 4 nurses who are required to have at least two years of prior ICU experience / comprehensive CCRU in-service training.	24-hours/7-days
Aslaner, 2015 [51]	Ankara, Turkey	Tertiary	ED-ICU	Eight-bed ED-ICU within Emergency Department with the same or similar staffing, monitoring, and capability for therapies as an ICU. Critical care such as invasive and non-invasive mechanical ventilation, invasive monitoring, use of vasoactive drugs, weaning, haemodialysis, paracentesis, and thoracentesis, were provided.	emergency medicine specialist, senior resident, nurses.	not given
Tseng, 2015 [52]	Taoyuan, Taiwan	Tertiary	ED-ICU	ICU located in the ED. The ED-ICU only admitted ED patients and when specialist physicians deemed critical care indicated but the respective ICU had no vacant bed.	Dual board-certified emergency medicine-critical care physician	not given
Schober, 2011 [53]	Vienna, Austria	Tertiary	ED-ICU (Acute Care Unit)	Two critical care resuscitation units with a total of 7 ICU beds admitting non-trauma critically ill patients for both resuscitation and subsequent critical care.	Intensivists and ICU-trained nurses as ED staff	24-hours/7-days
Piagnerelli, 2009 [54]	Brussels, Belgium	Tertiary	Shock room	Shock room with 4 beds located between the ED and the ICU used to stabilise all acutely ill patients in the hospital, both from outside and inside the hospital. 1 fully equipped ICU bed is always ready and reserved for critically ill patients. For patients admitted via the ED, admission to the shock room is determined using a triage system run by the ED team. For patients admitted from within the hospital or another hospital, decisions to admit to the shock room are made by the senior intensivist.	Managed jointly by ED and ICU physician staff. The shock room nursing staff is composed of ED and ICU nurses (two dedicated nurses for the four beds).	24-hours/7-days
Loria-Castellanos, 2006 [55]	Mexico City, Mexico	Secondary	Resuscitation Unit (RU)	Resuscitation unit within ED designed for assessment, evaluation, and initial treatment for very ill medical, surgical or trauma patients, that serves as the designated area in which to perform emergency procedures.	ED physician staff composed of 75 physicians (30 emergency physicians, 8 internal medicine physicians, 6 surgeons, 17 paediatricians, and 14 family medicine physicians)	24-hours/7-days
Brunette, 1999 [56]	Minneapolis, USA	Tertiary	Resuscitation Room	Resuscitation room in the ED.	ED physicians	24-hours/7-days
Bur, 1997 [57]	Vienna, Austria	Tertiary	ED-ICU (Acute Care Unit)	Two critical care resuscitation units with a total of 7 ICU beds admitting non-trauma critically ill patients for both resuscitation and subsequent critical care.	Intensivists and ICU-trained nurses as ED staff	24-hours/7-days
Hickey, 2020 [58]	New York, USA	Quaternary	ED-ICU	Creation of a temporary ED-based ICU during the coronavirus disease 2019 (COVID-19) pandemic. ED-ICU consisted of 13 critical care bays and 14 step-down beds.	Full-time support by dedicated emergency medicine attending. 6 dual-boarded emergency physicians in both emergency medicine and critical care medicine. 5 nurses with an additional three nurses 12 h per day with staggered shifts during peak day hours, 1 respiratory therapist per shift.	24-hours/7-days
Leith, 2020 [59]	Ann Arbor, USA	Quaternary	ED-ICU	see Gunnerson, 2019 [42]		
Smith, 2016 [60]	New York, USA	Acute care hospital / Tertiary	Emergency Intensive Care Unit (EICU)	EICU as a physically separate, 10-bed unit delivering emergency critical care adjacent to a freestanding ED.	Emergency intensivist staffing per American College of Critical Care Medicine (ACCM) requirements for Level II critical care centres. Emergency critical care–trained physicians, Critical care fellowship-trained Emergency Medicine physicians, Emergency Medicine board- certified physicians. 1 nurse manager with extensive emergency critical care experience. Respiratory care therapists, Critical care pharmacy and pharmacist services.	24-hours/7-days
Haas, 2020 [61]	Ann Arbor, USA	Quaternary	ED-ICU	see Gunnerson, 2019 [42]		
Haas, 2020 [62]	Ann Arbor, USA	Quaternary	ED-ICU	see Gunnerson, 2019 [42]		
Corrêa da Costa Ribeiro, 2018 [63]	Sao Paulo, Brasil	Tertiary	ED-ICU	ED-ICU within ED with 17 critical care beds.	ED-ICU physician	Not given
Chang, 2021 [64]	Taipei, Taiwan	Tertiary	Emergency Intensive Care Unit (EICU)	EICU is a 13-bed ICU within the ED. Acutely and critically ill patients who are not admitted to a specialized ICU are treated after initial ED resuscitation and stabilisation.	Not given	Not given
**Placement of critical care staff in the ED**						
Mitarai, 2023 [65]	Stanford, USA	Tertiary	ED-based intensivist and ICU nurse	Initial evaluation and resuscitation of critically ill medical patients provided by ED team. ED attending decided to activate the ED-based ICU attending, who evaluated the patient and decided on ICU admission. In case of ICU admission, ED-based intensivist and ICU nurse (together with ED nurse) provided critical care in the ED.	Dual board-certified emergency medicine-critical care physician and ICU nurse	Monday to Friday, 2.00 pm to midnight
Tuttle, 2023 [66]	Dallas, USA	Tertiary	ED-based medical ICU team	see Tuttle, 2022 [67]		
Tuttle, 2022 [67]	Dallas, USA	Tertiary	ED-based medical ICU team	ED physicians initially evaluated all critically ill medical patients and (based on consultation criteria) decided whether patients required medical ICU team evaluation. The team then provided diagnostic and therapeutic procedures as well as admission orders. In addition, the medical ICU team reviewed all ED patients awaiting ICU admission at 10.00 am.	Pulmonary and critical care physician, advanced practitioner provider, as well as pulmonary and critical care fellow. In addition, on a rotational daily basis, the ED nursing staff was supported by an ICU nurse.	10.00 am to 10.00 pm
Jayaprakash, 2022 [68]	Detroit, USA	Quaternary	ED-based critical care consultation service	Consultations of the ED-based critical care consultation service initiated by the ED physician according to suggested criteria indicating critical illness in ED patients or number of ICU boarders (e.g., if ≥ 2)	Board-eligible/certified physicians in both emergency and critical care medicine with clinical practices based on the ED and ICU	Monday to Friday 2.00 to 10.00 pm
Nesbitt, 2021 [69]	Stanford, USA	Tertiary	ED-based ICU nurse	As soon as critical illness became evident, ED-based ICU nurses were involved to assist the ED nurse to take care of critically ill patients (even before an ICU admission was decided). They implemented ICU bundles of care and assured a nurse: patient ratio of no greater than 1:2.	ED/ICU-dually trained nurses. All critically ill ED patients were treated by ED physicians until ICU admission.	Approximately 3 days per week.
**Deployment of critical care teams to the ED**						
Klotz, 2023 [70]	Maryland, USA	Network of 4 community hospitals and free-standing EDs	Emergency Medicine Stabilisation Team (EMSTAT)	A physician and nurse on-call ED model to provide improved care to critically ill patients requiring resuscitation when an ICU bed is not immediately available. EMSTAT employs a “go-team” of providers on call to support ICU patients boarding in rural EDs who destabilise or require high resource utilization.	A team of emergency physicians and nurses. One 12-hour shift was staffed by 1 emergency physician and 1 nurse. Emergency physicians either have a strong interest in critical care or are dually boarded in emergency medicine/critical care.	On call from 12 pm to 12 am on Monday through Friday.
Engebretsen, 2021 [71]	Oslo, Norway	Tertiary	Multi-disciplinary emergency response team	Multidisciplinary team called to all critically ill general medical patients in ED (according to defined criteria or a NEWS2-Score ≥ 5 points), who managed the patient in the ED and decided on subsequent ICU admission	Team led by internal medicine registrar and included anaesthesia registrar, nurse anaesthetist, phlebotomist, radiographer, and (if need) registrars from other specialties	24-hours/7-days
Elliot, 2015 [72]	Newark, USA	Tertiary	Medical ICU alert team dispatched to the ED	ED physicians activated the Medical ICU alert team, who expedited ICU admission or assumed total responsibility for the patient’s care in the ED	Medical ICU alert team consisted of an ICU nurse and physician assistant who provided care under supervision of an ICU attending	Monday to Thursday 7.00 am to 11 pm and Friday 7.00 am to 7.00 pm
Jensen, 2015 [73]	Holbaek, Denmark	Secondary	Emergency Team alert system	Activation of an alert system eliciting an emergency team call (“orange/red team call”) to facilitate further personnel resources from other hospital departments. Red emergency team calls are elicited prior to ambulance arrival by the senior ED physician.	Orange emergency team call: 1 consultant anaesthesiologist and 1 attending medical/surgical physician join the ED team Red emergency team call: 1 consultant anaesthesiologist, 1 nurse anaesthetist and 1 attending internal medicine physician join the ED team	not given
Christensen, 2011 [74]	Copenhagen, Denmark	Tertiary	Multi-disciplinary Team Reception protocol	Implementation of a multi-disciplinary team reception of critically ill and severely injured patients at the ED, termed “emergency call” and “trauma call”	Emergency call: ED nurse(s), anaesthesiologist, anaesthes-iologist trainee, nurse anaesthetist, medical doctor(s), secretary, hospital porter, two persons from radiology Trauma call: 2 ED nurses, anaesthesiologist, anaesthes-iologist trainee, nurse anae-sthetist, on-call orthopaedic surgeon, secretary, hospital porter, 2 persons from radiology	not given
**Telemedical support of ED staff by critical care team**						
Kadar, 2019 [75]	Chicago, USA	Tertiary	Telemedical support by medical ICU team	Four ED rooms equipped with mobile telemonitoring carts providing audiovisual monitoring and two-way communication. As soon as decision was made for medical ICU admission, telemedical support was initiated.	Care until ICU admission was telemedically guided by eICU intensivist and eICU nurse. Hands-on care (e.g., procedures, drug therapy) in the ED was provided by a hospital intensivist, ED nurse, and ED physician.	24-hours/7-days
Machado, 2018 [76]	Columbus, USA	Tertiary	Telemedical support by ICU team	An ED sepsis alert is generated when an ED patient fulfils sepsis criteria. Responders to the alert include an ED physician, a pharmacist, nurse and an X-ray technician (if available). The ED physician places orders based on the sepsis protocol. An eICU cart is connected to the patient and an eICU intensivist is contacted.	eICU intensivist reviews the care and makes changes or places additional orders as needed.	not given
Harvey, 2017 [77]	Charleston, USA	Fifty-two rural health-care settings	Telemedical consultation	Mobile telemedicine carts and peripheral examination devices to provide paediatric critical care consultations to ill patients presenting to rural hospitals	Paediatric critical care subspecialists for remote consultation, management, and facilitation of transfer	not given
Dharmar, 2013 [78]	Sacramento, USA	Five rural EDs	Telemedical consultation	On demand consultations by a paediatric critical care physician from an academic children’s hospital using dedicated videoconference systems	Telemedicine consultations involved live, interactive audiovisual communications between the ED physician, the paediatric patient, the ED nurse, the parent/guardian, and the consulting paediatric critical care physician	24-hours/7-days
Heath, 2009 [79]	Burlington, USA	Ten rural EDs	Telemedical consultation	Telemedical contact by the attending paediatric intensivist following a request for transport or consultation. Telephone lines and hardware-based dedicated videoconference systems were installed in the rural EDs, the paediatric ICU office, and the homes of the three participating paediatric intensivists	Three paediatric intensivists located at the referral hospital, a tertiary level 1 trauma centre	24-hours/7-days
**Implementation of protocols to accelerate ICU admission**						
Kim, 2019 [80]	Busan, South Korea	Tertiary	ICU admission protocol	ED physicians resuscitated critically ill ED patients and alerted the intensivist when ICU admission was deemed necessary.	Full-time Intensivist deciding on the ICU admission priority (level I, immediate admission; level III, delayed admission).	not given
Durie, 2017 [81]	Melbourne, Australia	Tertiary	“Code ICU” protocol	ED staff determined likelihood of ED patient requiring ICU bed and activated “code ICU”, which led to ICU registrar reviewing patient within 30 min expediting ICU transfer.	ICU registrar, who discusses patient with ICU consultant and ICU access nurse.	not given
McCoy, 2015 [82]	New Brunswick, USA	Tertiary	medical ICU admission protocol	Facilitated direct communication between ED physician and critical care attending or fellow. If ICU admission was warranted, a bed was requested and bed assignment began immediately. If no ICU bed was available, the medical ICU resident saw patient and wrote orders within an hour of the call.	ED physician, who directly contacted critical care attending or fellow to discuss critically ill patients.	not given

ED, emergency department; ED-ICU, emergency department-based intensive care unit; eICU, electronic (telemedical) intensive care unit; ICU, intensive care unit; NEWS2, National Early Warning Score 2; ResCCU, Resuscitation and Critical Care Unit; RU, resuscitation unit; USA, United States of America

**Fig. 4:**
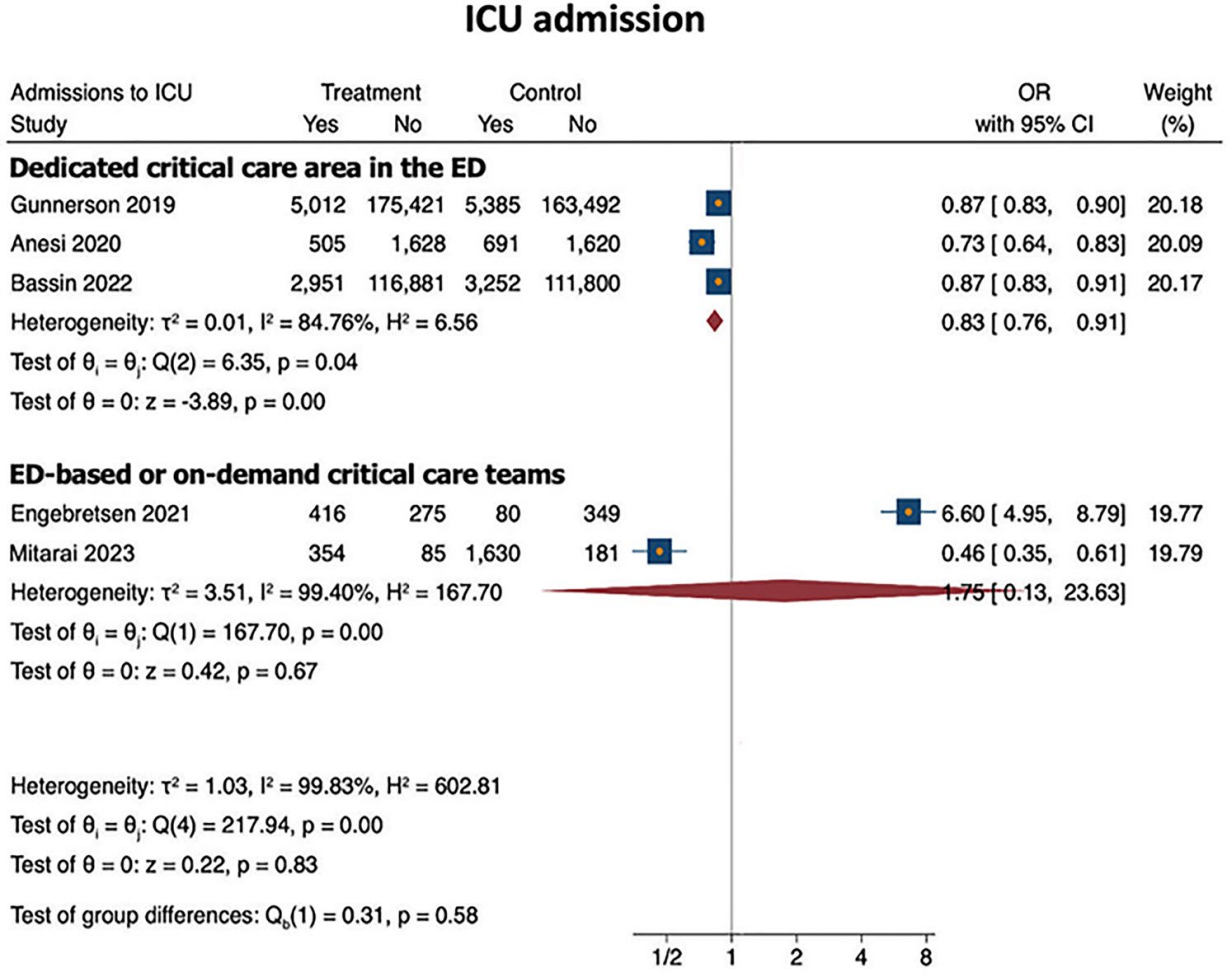
Pooled effects of different organisational model categories to deliver critical care in the emergency department on intensive care unit admission of emergency department patients. ED, emergency department; ED-ICU, intensive care unit located in the emergency department; ICU, intensive care unit

**Fig. 5:**
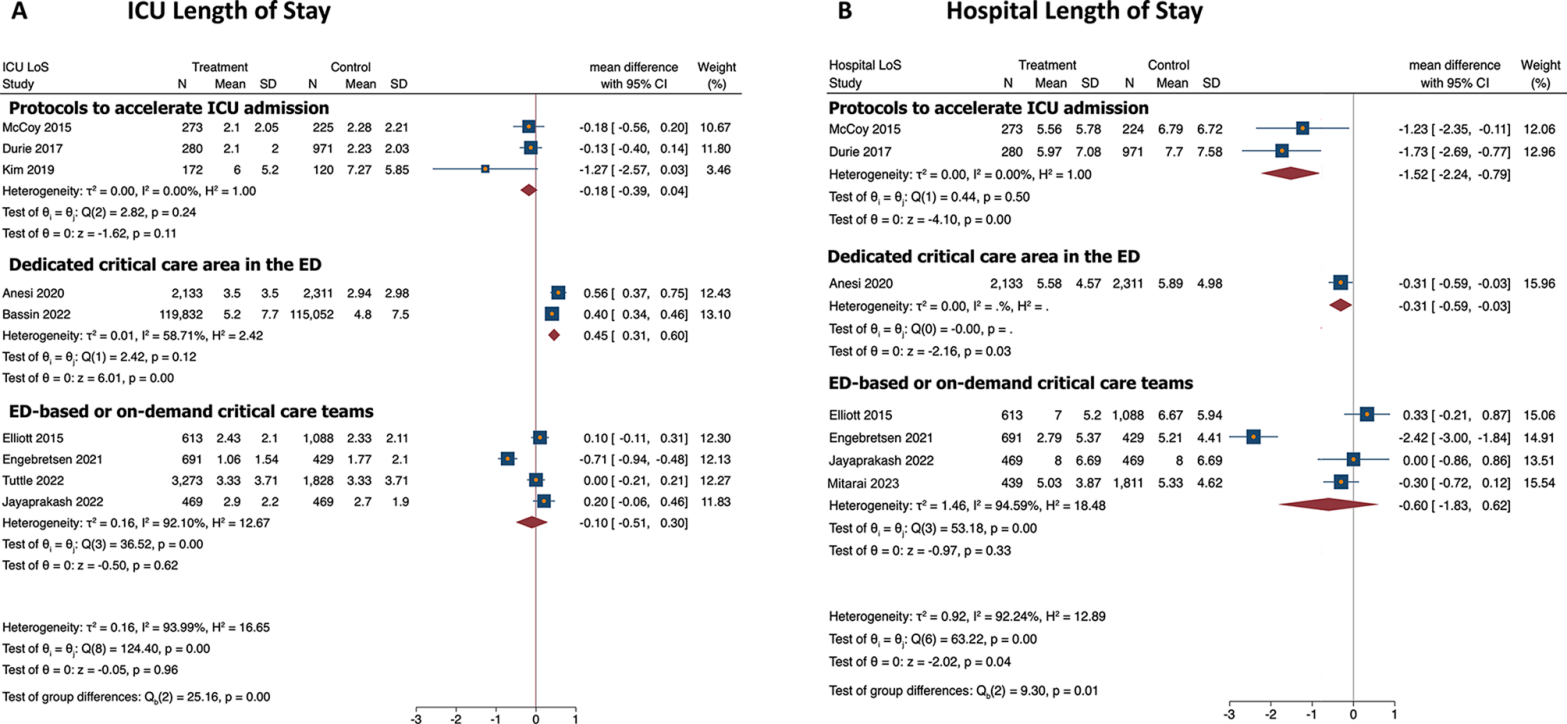
Pooled effects of different organisational model categories to deliver critical care in the emergency department on the LOS in the intensive care unit (**A**) and hospital (**B**) of emergency department patients. ED, emergency department; ED-ICU, intensive care unit located in the emergency department; ICU, intensive care unit

Compared to usual care, organisational models to deliver critical care in the ED had varying effects on ICU admission rates as well as hospital and ICU LOS. Five studies reported ICU admission rates and were included in the quantitative synthesis of this outcome. ICU admission rates together with the corresponding numerators and denominators for each included study are provided in Electronic Supplementary Table E2. Dedicated critical care areas in the ED decreased ICU admission rates [OR 0.83 (0.76–0.91), *I²*=84.7%, *p* < 0.001; analysis including adjusted estimates OR 0.82 (0.76–0.89), *I²*=80.2%, *p* < 0.001] (Fig. 4), but increased ICU LOS [0.45 (0.31–0.6) days, *I²*=58.7%, *p* < 0.001] (Fig. 5A) and decreased hospital LOS [-0.31 (-0.59 to -0.03) days, *p* = 0.03] (Fig. 5B). ED-based or on-demand critical care teams were not associated with consistent effects on ICU admission rates, ICU LOS or hospital LOS, respectively, as effect estimates varied substantially across studies. Protocols aimed at accelerating ICU admission were not associated with a reduction in ICU LOS [-0.18 (-0.39 to 0.04), *I²*=0%, *p* = 0.11] but with a shorter hospital LOS [-1.52 (-2.24 to -0.79), *I²*=0%, *p* < 0.001] (Fig. 5).

Three studies reported costs of care associated with organisational models to deliver critical care in the ED [ED-ICU, *n* = 1 [23]; ED-based critical care team, *n* = 1 [67]; protocol to accelerate ICU admission, *n* = 1 [82]]. The pooled mean difference in reported costs of care between organisational models to deliver critical care in the ED and usual care was − 551 USD (95% CI, -12,118 to + 11,017 USD) per patient (*I²*=98.6%, *p* = 0.86). Heterogeneity, as indicated by the *I²*-statistic, was high for all analyses.

Sensitivity analyses of studies categorised by their risk of bias indicated robust results of the main analyses (Electronic Supplementary Material Table E3). The level of evidence supporting the results of the quantitative meta-analysis was graded as low to very low.

## Discussion

This systematic review of the literature revealed five published organisational model categories to deliver critical care in the ED: (1) dedicated critical care areas in the ED; (2) placement of critical care staff in the ED; (3) deployment of critical care teams to the ED in case a critically ill patient was identified; (4) telemedical support of ED staff by critical care teams; and (5) implementation of protocols to accelerate ICU admission. Although each model could be classified into one of the five categories, certain overlaps in delivering critical care in the ED might still exist. Of all organisational models, dedicated critical care areas in the ED were by far the most frequently published model category. ICUs embedded in the ED, operating on a 24-hours/7-days basis, made up most of these dedicated critical care areas. Some ED-ICUs also included resuscitation areas, thereby constituting the entire geographic area of an ED, where critically ill patients are cared for. Further ED areas equipped and staffed to care for critically ill patients were CCRUs and resuscitation rooms. The published model of a CCRU resembles the ED-ICU concept but allows for both resuscitation and time-limited delivery of critical care to ED patients or those following inter-hospital transfer. The CCRU model has also recently been referred to as Critical Emergency Medicine Unit [83]. It is notable that the majority of EDs reporting critical care delivery models were staffed by critical care-trained physicians and nurses. Remarkably, almost half of physicians working in dedicated critical care areas in the ED were reported to be dually trained in emergency and critical care medicine.

The majority of publications on organisational models to deliver critical care in the ED originated from tertiary or quaternary medical centres. While this observed pattern may represent a publication bias, it is possible that organisational models to deliver critical care have been implemented less frequently in the EDs of lower-level care hospitals, potentially due to limited availability of adequately trained staff, medical equipment or financial resources. As indicated by the qualitative analysis of our study, it is conceivable that selected organisational models (e.g., deployment of critical care teams to the ED or telemedical support of ED staff by critical care teams) might be more commonly used in EDs with lower patient volumes than in those located in large academic centres. Furthermore, it is important to highlight that most organisational models, which have so far been published, focused exclusively on the care of critically ill adults in the ED. Only four studies meeting our inclusion criteria reported organisational models aimed at improving the care of critically ill children in paediatric EDs. Although the association between delays until ICU admission and adverse outcomes appears less pronounced in critically ill children [84] than adults [3–7], it is biologically plausible that timely delivery of critical care in the ED also improves the outcomes of critically ill children. The paucity of published organisational models to provide critical care in paediatric EDs may indicate under-implementation of such systems or a relevant lack of published evidence.

Previous research has indicated that early and adequate care of critically ill patients in the ED can reverse organ dysfunction, avoid ICU admission, and improve short- and long-term survival [2, 11]. Using a meta-analysis, we attempted to quantify the specific impact of single organisational models on patient-relevant outcomes. Our results suggest that dedicated critical care areas in the ED, such as ED-ICUs, are organisational models to deliver critical care in the ED capable of reducing the ICU admission rate and hospital LOS. The finding that critically ill ED patients, who required ICU admission, had a prolonged length of ICU stay might be explained by case-mix effects. ICU admission of critically ill ED patients with milder forms of organ dysfunction, or those who would have only required a short ICU stay, might have been prevented by sufficient stabilisation in the dedicated critical care areas in the ED. Consequently, the subset of critically ill ED patients eventually admitted to the ICU, likely represented a more severely ill cohort, requiring longer ICU treatment. Dedicated critical care areas in the ED investigated in this meta-analysis might therefore have functioned as a “pre-ICU sieve” and filtered patient streams. In line with these results, the finding that critical care delivery in the ED was cost-neutral appears reasonable, as resources within the ICU may have been preserved without compromising patient outcomes. In contrast, we found that no other organisational model category to deliver critical care in the ED was associated with a positive impact on patient-relevant outcomes. These findings generate the hypothesis that dedicated critical care areas may be particularly promising, but the low certainty of evidence precludes firm conclusions.

Several limitations need to be considered when interpreting the results of both the qualitative and quantitative analysis. First, as with all systematic reviews, some publications reporting organisational models to deliver critical care in the ED may have been missed. In addition, it is possible that certain models may have been implemented in clinical practice but have not been published in the international literature. Second, some centres published several studies repeatedly reporting the same critical care delivery model in the ED. This resulted in the over-representation of selected organisation models and was particularly relevant for ED-ICUs (i.e., eleven publications originated from one centre) as well as CCRUs (i.e., all seven publications originated from one centre). Although older studies were not included in the quantitative meta-analysis and therefore did not affect the pooled estimates, changes in clinical practice, monitoring standards, and care organisation over time may have influenced comparability within the descriptive synthesis. Third, we excluded 56 out of 67 studies from the quantitative meta-analysis, mostly because of study designs that rendered incomparable patient groups or overlapping observational periods. The resulting small number of studies limits the validity of the results of the quantitative meta-analysis, particularly in view of substantial heterogeneity of patient care and study-related characteristics. For example, available cost data were sparse and highly heterogenous, precluding reliable conclusions regarding cost effects. Fourth, some of the study interventions evaluated in the quantitative meta-analysis (e.g., establishment of dedicated critical care areas in the ED) precluded rigorous study designs such as randomized controlled trials. Accordingly, the overall level of evidence supporting the results of the quantitative meta-analysis was low to very low, and the results of the quantitative meta-analysis must hence be interpreted with particular caution.

Finally, consistent with Kümpers et al., emergency critical care should be characterised by disease severity, care intensity, and organ support rather than by location alone [85]. Accordingly, data should be interpreted cautiously, as they may reflect differences in case mix, frailty, comorbidities, treatment limitations, and ICU admission decisions rather than care quality in a specific location. Future studies should therefore assess not only structural aspects but also report clinical care intensity and therapeutic organ support more consistently.

## Conclusions

In conclusion, this systematic review revealed five different organisational model categories to deliver critical care in the ED. Dedicated critical care areas such as ED-ICUs, staffed by physicians and nurses dually trained in emergency and critical care, was the model category most frequently published. The quantitative meta-analysis suggested that compared with other CC-ED delivery models, these dedicated critical care areas in the ED may reduce both ICU admission rates and hospital LOS of critically ill ED patients.

## Supplementary Information

Below is the link to the electronic supplementary material.

Supplementary Material 1

Supplementary Material 2

## Data Availability

The datasets used and/or analysed during the current study are available from the corresponding author on reasonable request.

## Abbreviations

CC: Critical care
CCRU: Critical care resuscitation unit
CI: Confidence interval
ECCU: Emergency Critical Care Unit
EICU: Emergency Intensive Care Unit
ED: Emergency department
eICU: Electronic intensive care unit
ED-ICU: Intensive care unit located in the emergency department
GRADE: Grading of Recommendations Assessment, Development and Education
ICU: Intensive care unit
LOS: Length of stay
NEWS2: National Early Warning Score 2
ResCCU: Resuscitation and Critical Care Unit
RU: Resuscitation unit
