# Supplementary material for: Critical care delivery models in emergency departments: a systematic review of the literature and meta-analysis of related outcome effects
Source: Scand J Trauma Resusc Emerg Med. 2026 Jun 14;34:132. doi: 10.1186/s13049-026-01647-9 (PMC13439787; doi:10.1186/s13049-026-01647-9)
Supplement: Supplementary file 1 [file 13049_2026_1647_MOESM1_ESM.docx]

Electronic Supplementary Material to:

**Critical Care Delivery Models in Emergency Departments: A Systematic Review of the Literature and Meta-Analysis of Related Outcome Effects**

Matthias NOITZ, MD^1,2^; Steven A. ILKO, MD^3,4^; Martin W. DÜNSER, MD^1,2^; Wilhelm BEHRINGER, MD^5^; Brigitte WILDNER^6^; Harald HERKNER, MD^5^

^1^, Department of Anesthesiology and Critical Care Medicine, Kepler University Hospital GmbH, Johannes Kepler University Linz, Krankenhausstraße 9, 4020 Linz and Altenberger Strasse 69, 4040 Linz, Austria; ^2^, Medical Faculty, Johannes Kepler University, Linz, Austria; ^3^, Department of Emergency Medicine Center for Adult Critical Care, University of New Mexico, Albuquerque, New Mexico, USA; ^4^, Department of Anesthesia, University of Iowa Healthcare, Iowa City, Iowa, USA; ^5^, Department of Emergency Medicine, Medical University of Vienna, Vienna, Austria; ^6^, University Library, Medical University of Vienna, Vienna, Austria.

*Address for Correspondence:*
Matthias Noitz, MD; Department of Anesthesiology and Intensive Care Medicine, Kepler University Hospital GmbH, Johannes Kepler University Linz, Krankenhausstraße 9, 4020 Linz and Altenberger Strasse 69, 4040 Linz, Austria.Tel.: +43 5 7680 83 2158, Email: mnoitz@gmx.at

**Electronic Supplementary Material Table E1.** Search terms and session results for the systematic literature review used in EMBASE (https://www.embase.com/).

|  | **EMBASE search strategy** |  |
| --- | --- | --- |
| #83 | #82 NOT ('conference abstract':it OR review:it OR letter:it OR note:it OR editorial:it OR 'conference review':it OR 'short survey':it OR erratum:it) | 1727 |
| #82 | #37 AND (#38 OR #39 OR #40 OR #41 OR #42 OR #43 OR #44 OR #45 OR #46 OR #81) | 2543 |
| #81 | #66 NOT #80 | 5733888 |
| #80 | #67 OR #68 OR #69 OR #70 OR #71 OR #72 OR #73 OR #74 OR #75 OR #76 OR #77 OR #78 OR #79 | 4438933 |
| #79 | 'animal experiment'/de NOT ('human experiment'/de OR 'human'/de) | 2611585 |
| #78 | (rat:ti,tt OR rats:ti,tt OR mouse:ti,tt OR mice:ti,tt OR swine:ti,tt OR porcine:ti,tt OR murine:ti,tt OR sheep:ti,tt OR lambs:ti,tt OR pigs:ti,tt OR piglets:ti,tt OR rabbit:ti,tt OR rabbits:ti,tt OR cat:ti,tt OR cats:ti,tt OR dog:ti,tt OR dogs:ti,tt OR cattle:ti,tt OR bovine:ti,tt OR monkey:ti,tt OR monkeys:ti,tt OR trout:ti,tt OR marmoset*:ti,tt) AND 'animal experiment'/de | 1242876 |
| #77 | (databases NEAR/5 searched):ab | 70004 |
| #76 | 'update review':ab | 138 |
| #75 | 'we searched':ab AND (review:ti,tt OR review:it) | 51117 |
| #74 | review:ab AND review:it NOT trial:ti,tt | 1152837 |
| #73 | ('random cluster' NEAR/4 sampl*):ti,ab,tt | 1626 |
| #72 | 'random field*':ti,ab,tt | 2979 |
| #71 | nonrandom*:ti,ab,tt NOT random*:ti,ab,tt | 19209 |
| #70 | 'systematic review':ti,tt NOT (trial:ti,tt OR study:ti,tt) | 272363 |
| #69 | 'case control*':ti,ab,tt AND random*:ti,ab,tt NOT ('randomised controlled':ti,ab,tt OR 'randomized controlled':ti,ab,tt) | 21900 |
| #68 | 'cross-sectional study'/de NOT ('randomized controlled trial'/exp OR 'controlled clinical study'/de OR 'controlled study'/de OR 'randomised controlled':ti,ab,tt OR 'randomized controlled':ti,ab,tt OR 'control group':ti,ab,tt OR 'control groups':ti,ab,tt) | 375223 |
| #67 | ((random* NEXT/1 sampl* NEAR/8 ('cross section*' OR questionnaire* OR survey OR surveys OR database OR databases)):ti,ab,tt) NOT ('comparative study'/de OR 'controlled study'/de OR 'randomised controlled':ti,ab,tt OR 'randomized controlled':ti,ab,tt OR 'randomly assigned':ti,ab,tt) | 3228 |
| #66 | #47 OR #48 OR #49 OR #50 OR #51 OR #52 OR #53 OR #54 OR #55 OR #56 OR #57 OR #58 OR #59 OR #60 OR #61 OR #62 OR #63 OR #64 OR #65 | 6496680 |
| #65 | trial:ti,tt | 419504 |
| #64 | 'human experiment'/de | 656123 |
| #63 | volunteer:ti,ab,tt OR volunteers:ti,ab,tt | 287687 |
| #62 | (controlled NEAR/7 (study OR design OR trial)):ti,ab,tt | 463300 |
| #61 | assigned:ti,ab,tt OR allocated:ti,ab,tt | 501455 |
| #60 | ((assign* OR match OR matched OR allocation) NEAR/5 (alternate OR group OR groups OR intervention OR interventions OR patient OR patients OR subject OR subjects OR participant OR participants)):ti,ab,tt | 423442 |
| #59 | crossover:ti,ab,tt OR 'cross over':ti,ab,tt | 126735 |
| #58 | (parallel NEXT/1 group*):ti,ab,tt | 32933 |
| #57 | 'double blind procedure'/de | 215519 |
| #56 | ((double OR single OR doubly OR singly) NEXT/1 (blind OR blinded OR blindly)):ti,ab,tt | 280205 |
| #55 | (open NEXT/1 label):ti,ab,tt | 112254 |
| #54 | (evaluated:ab OR evaluate:ab OR evaluating:ab OR assessed:ab OR assess:ab) AND (compare:ab OR compared:ab OR comparing:ab OR comparison:ab) | 2852015 |
| #53 | compare:ti,tt OR compared:ti,tt OR comparison:ti,tt | 638562 |
| #52 | placebo:ti,ab,tt | 372329 |
| #51 | 'intermethod comparison'/de | 305927 |
| #50 | 'randomization'/de | 98807 |
| #49 | random*:ti,ab,tt | 2022659 |
| #48 | 'controlled clinical trial'/de | 442020 |
| #47 | 'randomized controlled trial'/exp | 800996 |
| #46 | 'major clinical study':ti,ab,kw,de | 5206240 |
| #45 | (comparative* NEAR/2 (study OR studies OR effectiveness)):ti,ab,kw,de | 1321459 |
| #44 | (longitudinal* NEAR/2 (study OR studies OR evaluation*)):ti,ab,kw,de | 272376 |
| #43 | ('cross sectional*' NEAR/2 (study OR studies OR design* OR research*)):ti,ab,kw,de | 700162 |
| #42 | (retrospective* NEAR/2 (study OR studies OR design*)):ti,ab,kw,de | 1684933 |
| #41 | ('case control*' NEAR/2 (study OR studies)):ti,ab,kw,de | 281169 |
| #40 | (prospective* NEAR/2 (study OR studies OR method*)):ti,ab,kw,de | 1148975 |
| #39 | (cohort* NEAR/2 (study OR studies OR analys*)):ti,ab,kw,de | 1231693 |
| #38 | (observation* NEAR/2 (study OR studies)):ti,ab,kw,de | 469047 |
| #37 | #34 OR #35 OR #36 | 6754 |
| #36 | 'critical care resuscitation unit*':ti,ab,kw | 42 |
| #35 | (icu:ti OR icus:ti OR 'intensive care*':ti OR 'critical care*':ti OR 'critical* ill*':ti) AND ('ed based':ti OR (((emergen* OR 'a and e' OR 'a-e') NEAR/2 (department* OR ward* OR unit* OR room*)):ti)) | 972 |
| #34 | #19 AND (#20 OR #21 OR #22 OR #23 OR #24 OR #25 OR #26 OR #27 OR #28 OR #29 OR #30 OR #31 OR #32 OR #33) | 4647 |
| #33 | (hybrid NEAR/3 (emergen* OR er OR ed OR icu*)):ti,ab,kw | 361 |
| #32 | 'resuscitation room management*':ti,ab,kw | 27 |
| #31 | (('critical care' OR 'intensive care') NEAR/3 (model* OR center* OR program* OR team* OR staff* OR solution* OR intervention* OR deliver* OR suppl*)):ti,ab,kw | 12081 |
| #30 | (emergency* NEAR/2 ('intensive care' OR 'critical care' OR 'critically ill' OR 'critical illness' OR icu* OR icus OR intervention*)):ti,ab,kw | 8821 |
| #29 | (('emergency department*' OR 'emergency room*' OR 'emergency medicine*' OR 'ed based' OR ed) NEAR/3 (team* OR staff) NEAR/2 ('intensive care' OR 'critical care' OR 'critically ill' OR 'critical illness' OR icu OR icus OR intervention*)):ti,ab,kw | 102 |
| #28 | (('emergency department*' OR 'emergency room*' OR 'emergency medicine*' OR 'ed based' OR ed) NEAR/3 ('intensive care' OR 'critical care' OR 'critically ill' OR 'critical illness' OR icu OR icus OR intervention*)):ti,ab,kw | 9334 |
| #27 | 'average volume assured pressure support':ti,ab,kw OR 'helmet continuous positive airway pressure':ti,ab,kw | 199 |
| #26 | 'nasal-cannula':ti,ab,kw OR 'nasal-prong*':ti,ab,kw OR 'nasal-tube*':ti,ab,kw OR 'nasal-catheter*':ti,ab,kw OR 'mouth to mouth':ti,ab,kw | 9274 |
| #25 | (('pressure support' OR 'positive pressure' OR noninvasive OR 'non invasive' OR helmet OR mask OR mouthpiece) NEAR/4 (ventilat* OR respirat*)):ti,ab,kw | 38128 |
| #24 | 'noninvasive ventilation'/exp OR 'pressure support ventilation'/exp | 30251 |
| #23 | 'patient monitoring'/exp OR monitoring*:ti,ab,kw OR telemonitoring*:ti,ab,kw OR telesurveillance*:ti,ab,kw OR neuromonitoring*:ti,ab,kw OR surveillance*:ti,ab,kw | 1387455 |
| #22 | rrt:ti,ab,kw | 12177 |
| #21 | dialysis:ti,ab,kw,de OR hemodialysis:ti,ab,kw,de OR haemodialysis:ti,ab,kw,de OR hemofiltration:ti,ab,kw,de OR hemodiafiltration:ti,ab,kw,de OR 'replacement therapy':ti,ab,kw,de | 447779 |
| #20 | 'renal replacement therapy'/exp | 248400 |
| #19 | #3 AND #18 OR (#12 AND #9) | 11990 |
| #18 | #13 OR #14 OR #15 OR #16 OR #17 | 450567 |
| #17 | 'critical care*':ti,kw OR 'critical* ill*':ti,kw | 77903 |
| #16 | 'intensive care*':ti,kw | 99143 |
| #15 | 'intensive care'/mj | 69515 |
| #14 | icu:ti,kw OR icus:ti,kw | 31452 |
| #13 | 'intensive care unit'/mj | 45670 |
| #12 | #10 OR #11 | 81120 |
| #11 | (((emergen* OR 'a and e' OR 'a-e') NEAR/2 (department* OR ward* OR unit* OR room*)):ti,kw) OR 'ed based':ti,kw | 77516 |
| #10 | 'emergency ward'/exp/mj | 46326 |
| #9 | #4 OR #5 OR #6 OR #7 OR #8 | 1251246 |
| #8 | 'critical care*':ti,ab,kw OR 'critical* ill*':ti,ab,kw | 167060 |
| #7 | 'intensive care*':ti,ab,kw | 299047 |
| #6 | 'intensive care'/de | 150368 |
| #5 | icu:ti,ab,kw OR icus:ti,ab,kw | 179801 |
| #4 | 'intensive care unit'/exp | 306480 |
| #3 | #1 OR #2 | 307093 |
| #2 | (((emergen* OR 'a and e' OR 'a-e') NEAR/2 (department* OR ward* OR unit* OR room*)):ti,ab,kw) OR 'ed based':ti,ab,kw | 254710 |
| #1 | 'emergency ward'/exp | 220900 |

**Electronic Supplementary Figure E1. Domain-level risk-of-bias assessment of studies included in the quantitative synthesis.**

D1, temporal precedence; D2, selection/allocation; D3, confounding; D4, intervention administration; D5, outcome measurement. Domain-level judgements were derived from the corresponding Joanna Briggs Institute risk-of-bias assessment items. Overall risk-of-bias judgements reflected the anticipated impact of domain-specific concerns on the validity of the effect estimate and were not assigned using a strict worst-domain rule.

**Electronic Supplementary Table E2.** ICU admission rates among studies included in the quantitative synthesis of ICU admission.

| **Organisational model** | **Study** | **ICU admissions (intervention)** | **Total (intervention)** | **ICU admission rate (%) intervention** | **ICU admissions (control)** | **Total (control)** | **ICU admission rate (%) control** |
| --- | --- | --- | --- | --- | --- | --- | --- |
| **Dedicated critical care area in the ED** | Gunnerson, 2019 | 5,012 | 180,433 | 2.8 | 5,385 | 168,877 | 3.2 |
|  | Anesi, 2020 | 505 | 2,133 | 23.7 | 691 | 2,311 | 29.9 |
|  | Bassin, 2022 | 2,951 | 119,832 | 2.5 | 3,252 | 115,052 | 2.8 |
| **ED-based or on-demand critical care teams** | Engebretsen, 2021 | 416 | 691 | 60.2 | 80 | 429 | 18.6 |
|  | Mitarai, 2023 | 354 | 439 | 80.6 | 1,630 | 1,811 | 90.0 |

ED, emergency department.

**Electronic Supplementary Material Table E3.** Sensitivity analyses of quantitative meta-analysis.

| **Outcome Variable** | **Risk of Bias** | **Dedicated critical care area  in the ED** | **ED-based or on-demand Critical Care Team** | **Protocols to accelerate  ICU admission** |
| --- | --- | --- | --- | --- |
| **Mortality** | **low** | 1.01 (0.96 to 1.05); 1 study | 0.86 (0.71 to 1.05); 2 studies | No studies extracted |
|  | **high** | 1.18 (0.98 to 1.42); 1 study | 1.07 (0.93 to 1.22); 3 studies | 1.25 (0.91 to 1.7); 3 studies |
| **ICU admission** | **low** | 0.87 (0.84 to 0.89); 2 studies | 0.46 (0.35 to 0.61); 1 study | No studies extracted |
|  | **high** | 0.73 (0.64 to 0.83); 1 study | 6.6 (4.95 to 8.79); 1 study | No studies extracted |
| **ICU LOS** | **low** | 0.40 (0.34 to 0.46); 1 study | 0.10 (-0.11 to 0.31); 1 study | No studies extracted |
|  | **high** | 0.56 (0.37 to 0.75); 1 study | -0.17 (-0.71 to 0.37); 3 studies | -0.18 (-0.39 to 0.04); 3 studies |
| **Hospital LOS** | **low** | No studies extracted | -0.01 (-0.62 to 0.61); 2 studies | No studies extracted |
|  | **high** | -0.31 (-0.59 to -0.03); 1 study | -1.23 (-3.6 to 1.14); 2 studies | -1.52 (-2.24 to -0.79); 2 studies |

ED, emergency department; ICU, intensive care unit; LOS, length of stay.
